# Eosinophilic granulomatosis with polyangiitis coexisting with multiple myeloma: independent entities or coexistence? A case report

**DOI:** 10.3389/fimmu.2026.1751926

**Published:** 2026-03-02

**Authors:** Longyan Qin, Yi Yin, Pengjia Wu, Aifei Zhang, Na Li, Jun Liu, Ling Huang, Jun Cao, Jiashun Zeng

**Affiliations:** 1 Department of Rheumatology and Immunology, The Affiliated Hospital of Guizhou Medical University, Guiyang, China; 2 Department of Radiology, Affiliated Hospital of Guizhou Medical University, Guiyang, China; 3 Department of Laboratory Medicine, Guizhou Provincial People’s Hospital, Guiyang, China; 4 Department of Hematology, Guizhou Provincial People’s Hospital, Guiyang, China

**Keywords:** eosinophil, eosinophilic granulomatosis with polyangiitis, mepolizumab, multiple myeloma, plasma cell neoplasm

## Abstract

Eosinophilic granulomatosis with polyangiitis (EGPA) represents a systemic necrotizing vasculitis characterized by prominent peripheral eosinophilia. Concomitant plasma cell dyscrasias in the context of EGPA remain exceedingly rare in clinical literature. This paper presents a case of a 49-year-old female patient with recurrent diarrhea, rash, fever, and wheezing. The patient was initially diagnosed with multiple myeloma (MM) due to significant elevation of M protein and immature plasma cells in the bone marrow. However, chemotherapy failed to alleviate her condition, while standardized EGPA treatment achieved disease control. This case of EGPA with clinical manifestations of MM prompts us to consider whether the clinical presentation should follow Occam’s razor or Hickam’s dictum. Ultimately, we hypothesize that EGPA-induced eosinophil (EOS) perturbs the bone marrow hematopoietic niche. Through the Th2-mediated cytokine milieu, this microenvironment may trigger a reactive, “pseudo-malignant” expansion of plasma cells.

## Introduction

EGPA is an ANCA-associated vasculitis characterized by eosinophilia and involvement of small vessels and multiple systems. Clinical manifestations include fever, rash, asthma, weight loss, myalgia, neuropathy, pulmonary infiltrates, and/or nasal abnormalities (1).MM is a malignant proliferative plasma cell neoplasm characterized by the presence of abnormal clonal plasma cells in the bone marrow, accompanied by elevated levels of monoclonal immunoglobulins or light chains (2). Both EOS and plasma cells originate from multipotent hematopoietic stem cells. Many lymphoid malignancies(e.g., T-cell and B-cell lymphomas and Hodgkin lymphoma) can present with eosinophilia (3). However, cases of eosinophilia associated with plasma cell disorders are relatively rare (4, 5), and the coexistence of EGPA and plasma cell disorders is even rarer. This paper reports a case of EGPA with clinical manifestations related to MM and preliminarily explores the logical relationship between these two diseases. Written informed consent was obtained from the patient for this case report, and all diagnostic and therapeutic data collection and usage complied with the ethical standards of the Declaration of Helsinki.

## Case report

A 49-year-old female patient presented with a 7-year history of relapsing diarrhea, cutaneous rash, and intermittent pyrexia.

**Figure 1:**
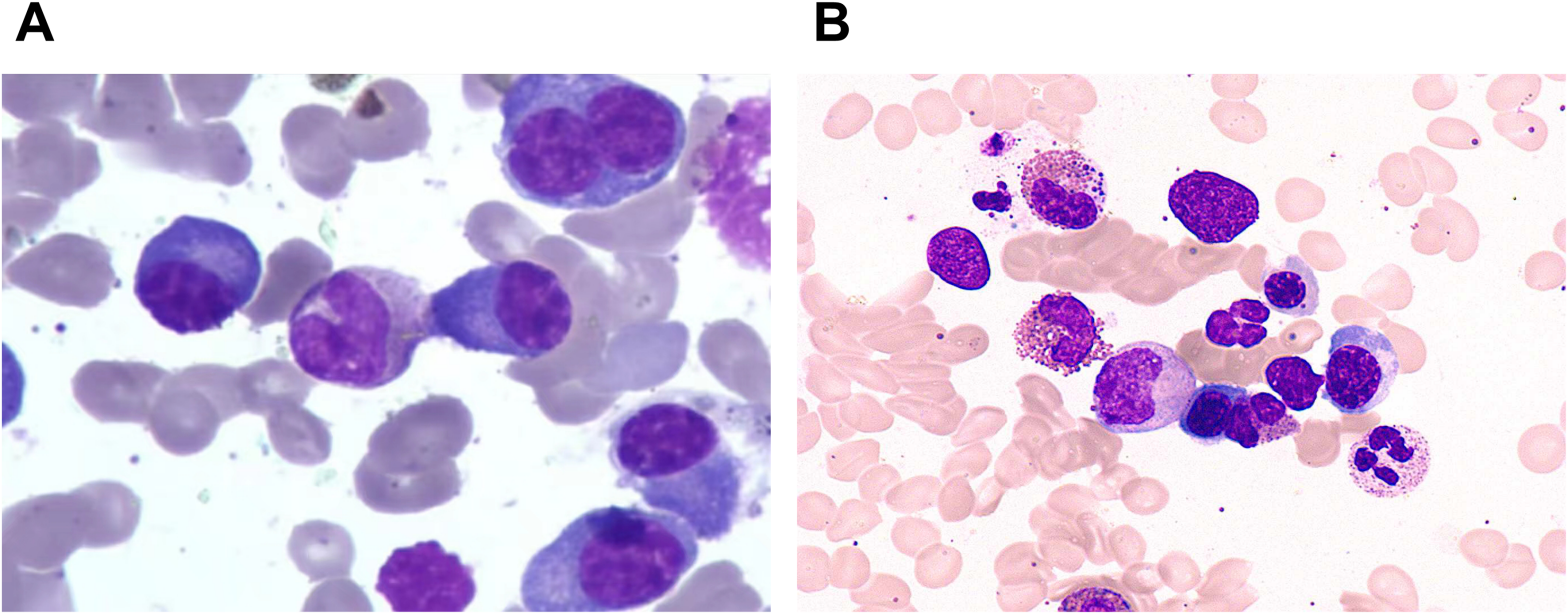
Bone marrow staining **(A)** Numerous plasma cells and scattered eosinophils (Wright-Giemsa stain, ×1000, Scale bar = 10 μm) **(B)** Numerous eosinophils (Wright-Giemsa stain, ×1000, Scale bar = 10 μm).

At the initial diagnosis seven years ago, the patient presented with intermittent dull pain in the upper abdomen accompanied by yellow watery stools approximately 10 times daily, scattered rash over the body, fever ranging from 37.7°C to 39.5°C, fatigue, and loss of appetite. Laboratory tests at another hospital revealed: hemoglobin (Hb)80g/L (reference:114-163g/L), EOS 2.07×10^9^/L (reference:0.02-0.52×10^9^/L), EOS% 25.3% (reference: 0.4-8%); 24-hour urinary protein 350 mg/24h; urinary κ light chain 387 mg/L (reference: 0–51 mg/L), urinary λ light chain <50 mg/L (reference: 0–50 mg/L); serum κ light chain 37100 mg/L (reference: 1380–3750 mg/L), λ light chain 11800 mg/L (reference: 930-2420mg/L), serum free κ light chain 33.1 mg/L (reference: 0.33-1.96 mg/L), serum free λ light chain 14.5 mg/L (reference: 0.57-2.63 mg/L). Albumin 19.2 g/L (reference: 40–55 g/L), total bilirubin (TBiL) 185.8 μmol/L (reference: 40-55 μmol/L), direct bilirubin (DBiL) 49.7 μmol/L (reference: 0-0.86 μmol/L), creatinine (Cr) 127.6 μmol/L(reference: 44-97 μmol/L), β_2_-microglobulin 7.84 mg/L (reference: 1.0-3.0 mg/L), LDH 243 U/L (reference: 109–248 U/L). Immunoglobulin G (IgG) 47.46 g/L (reference range: 7.0-16.0 g/L), immunoglobulin A(IgA) 1.2 g/L (reference: 0.7-4.0 g/L), immunoglobulin E (IgE) 2266.3 IU/mL (reference: 0–100 IU/mL), immunoglobulin G4 (IgG4) 22.25 g/L (reference: 0.08-1.4 g/L). Complement C3 0.78 g/L (reference: 0.8-1.8 g/L), C4 0.14 g/L (reference: 0.1-0.4 g/L). Serum protein electrophoresis showed M protein 26.9%, Alb 36.5%, α_1_ globulin 3.5%, α_2_ globulin 10.0%, β globulin 9.1%, γ globulin 14%, “M” 26.9%. Stool routine, blood calcium, antineutrophil cytoplasmic antibodies (ANCA), antinuclear antibodies (ANA), autoimmune liver antibodies, antiphospholipid antibodies, and parasite tests were all negative. Immunofixation electrophoresis revealed abnormal biclonal bands of IgG+κ and IgG+λ. Bone marrow aspirate demonstrated aberrant plasma cell proliferation with 23% immature plasma cells and 5.5% mature plasma cells, along with significant EOS (**Figure 1A**). Biopsy revealed active bone marrow hyperplasia, increased eosinophils, and plasma cells with scattered distribution, no focal or diffuse infiltration, and MF-0 grade. Bone marrow flow cytometry immunohistochemistry showed CD3^+^, CD5^+^, CD20^+^, PAX5^+^, CD38^+^, CD138^+^, Kappa^+^, Lambda^+^, co-expression of both light chains, no monotypic light chain restriction, CD56^-^, CD30^-^. Cytogenetic evaluations, including FISH and karyotyping, yielded unremarkable results. Liver biopsy showed chronic active hepatitis with eosinophilic and plasma cell infiltration and IgG4 (+2/HPF), without storiform fibrosis or obliterative phlebitis.

Bone marrow aspiration results indicated that immature and mature plasma cells accounted for 28.5%, immunofixation electrophoresis confirmed IgG-κ+IgG-λ biclonal M protein, and serum protein electrophoresis showed M protein accounting for 26.9%. After discussion among multiple hematologists, the patient was diagnosed with MM: IgG-λ+IgG-κ. End-organ damage met the CRAB criteria for MM, with Hb 80g/L, Cr 127.6 μmol/L, serum β_2_-microglobulin 7.84 mg/L, and albumin 19.2 g/L, meeting the ISS stage III criteria. After completing two cycles of the VRD chemotherapy regimen (bortezomib 1.3 mg/m² on days 1, 4, 8, and 11 combined with lenalidomide 10 mg on days 2–22 and dexamethasone 40 mg on days 1-4, 9-12, and 17-20), follow-up showed M protein turned negative, and the bone marrow plasma cell proportion decreased to 3%. After nine additional cycles of the same regimen, the patient developed glove-and-stocking paresthesia (suspected to be caused by bortezomib), and the regimen was adjusted to BD (lenalidomide + dexamethasone) chemotherapy. Over the next six years, the patient continued to experience intermittent diarrhea, rash, fever, and wheezing, with body temperature fluctuating between 37.2°C and 39.0°C, and the nature of diarrhea remained unchanged. Multiple adjustments to the chemotherapy regimen, including seven cycles of the IRD regimen (ixazomib + lenalidomide + dexamethasone), followed by daily oral lenalidomide 10 mg maintenance therapy, failed to control the disease. Blood tests consistently showed eosinophil counts (0.77-2.87)×10^9^/L, EOS% 9.4%-20.3%, hemoglobin 80–114 g/L, and mild liver and kidney dysfunction. Twelve repeated bone marrow examinations consistently showed eosinophilia. Two immunofixation electrophoresis tests revealed abnormal biclonal bands of IgG+κ and IgG+λ, with no other abnormalities.

The patient sought treatment at another hospital, where MPO was suspected to be positive. The physicians there considered the possibility of connective tissue disease due to the patient’s multi-system involvement. Bone marrow findings of M protein, reduced hemoglobin, and mildly elevated creatinine were attributed to connective tissue disease, leading to a diagnosis of smoldering MM. Treatment with oral methylprednisolone 60 mg daily and azathioprine 0.2 g daily initially improved diarrhea, rash, and wheezing symptoms significantly, with no fever. However, when the methylprednisolone dose was gradually reduced to 16 mg, the symptoms recurred. One month before admission to our center, the patient experienced bloody diarrhea, fever (38.6°C), and a weight loss of approximately 10 kg.

### Past medical history

The patient had a history of asthma for nine years, treated with regular salmeterol/fluticasone inhalation but with frequent exacerbations. She also had a history of urticaria for eight years, treated with antihistamines and corticosteroids. Five years ago, she underwent cholecystectomy for gallstones.

### Physical examination and auxiliary tests

Blood pressure was 153/98 mmHg. Multiple pea-sized lymph nodes were palpable in both axillae. A soft liver was palpable 5cm below the right costal margin, with tenderness in the right upper abdomen. To further verify the MM diagnosis, the following tests were performed: WBC 8.14×10^9^/L, EOS 2.54×10^9^/L (reference: 0.02-0.52×10^9^/L), EOS% 30% (reference: 0.4-8.0%), Hb 97 g/L (reference: 115-150g/L); 24-hour urinary protein 290 mg/24h, alanine aminotransferase 56.9 U/L (reference: 7-40U/L), albumin 32.8 g/L (reference: 40-55g/L), TBiL 33.9 μmol/L (reference: ≤23μmol/L), DBiL 18.00 μmol/L (reference: ≤8μmol/L), γ-GGT 85 U/L (reference: 7-45U/L), Cr 132.5 μmol/L (reference: 41-96μmol/L), β_2_-microglobulin 3.69 mg/L (reference: 1.0-3.0 mg/L), LDH 1385 U/L (reference: 120–250 U/L). IgG 40 g/L (reference: 8.6-17.4 g/L), IgA 1.2 g/L (reference: 1.0-4.2 g/L), IgM 2.3 g/L (reference: 0.5-2.8 g/L), IgE 1518.85 IU/mL (reference: 0–100 IU/mL), complement C3 0.411 g/L (reference: 0.7-1.4g/L), C4 0.0167 g/L (reference: 0.1-0.4g/L). Urinary κ light chain 4610 mg/L (reference: ≤18.5 mg/L), urinary λ light chain 64.4 mg/L (reference: ≤50 mg/L), serum κ light chain 34400 mg/L (reference: 1700–3700 mg/L), serum λ light chain 15500 mg/L (reference: 900–2100 mg/L), serum free κ light chain 127.98 mg/L (reference:0.33-1.96 mg/L), serum free λ light chain 123.15 mg/L (reference: 0.57-2.63 mg/L). Serum protein electrophoresis: Alb 34%, α_1_ globulin 3.5%, α_2_ globulin 7.7%, β globulin 5.9%,γ globulin 48.9%. Echocardiography showed a small amount of pericardial effusion. Abdominal ultrasound indicated hepatosplenomegaly. Parotid ultrasound was normal. Superficial organ color Doppler showed multiple enlarged lymph nodes in the bilateral neck and axillae, with the largest measuring 19×10 mm. Bone marrow aspiration revealed EOS (19%) (**Figure 1B**).

**Figure 2:**
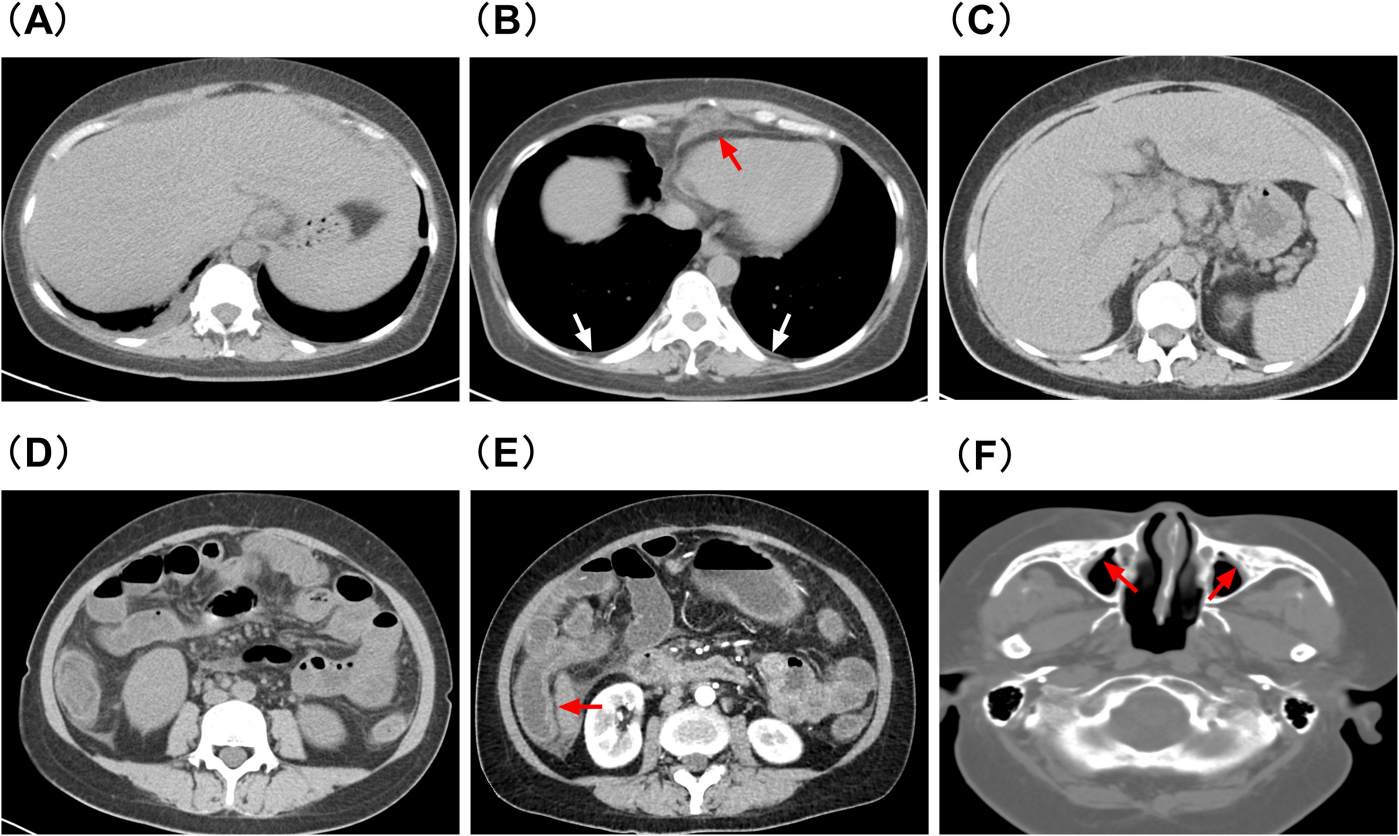
**(A–D, F)** are plain CT scans, and **(E)** is contrast-enhanced CT. **(A)** Bilateral pleural effusion **(B)** Pleural thickening (white arrow) and pericardial effusion (red arrow) **(C)** Hepatomegaly, splenomegaly, and multiple enlarged lymph nodes **(D)** Multiple abdominal exudates with blurred fat spaces and flocculent high-density shadows **(E)** Thickened ascending colon wall (red arrow) and exudation around the transverse colon **(F)** Bilateral maxillary sinus mucosal thickening (red arrow).

Chest and abdominal CT showed pleural effusion, pericardial effusion, hepatosplenomegaly, and significant mesenteric exudation (**Figures 2A–E**). Sinus CT revealed inflammatory changes in the maxillary sinuses (**Figure 2F**).

**Figure 3:**
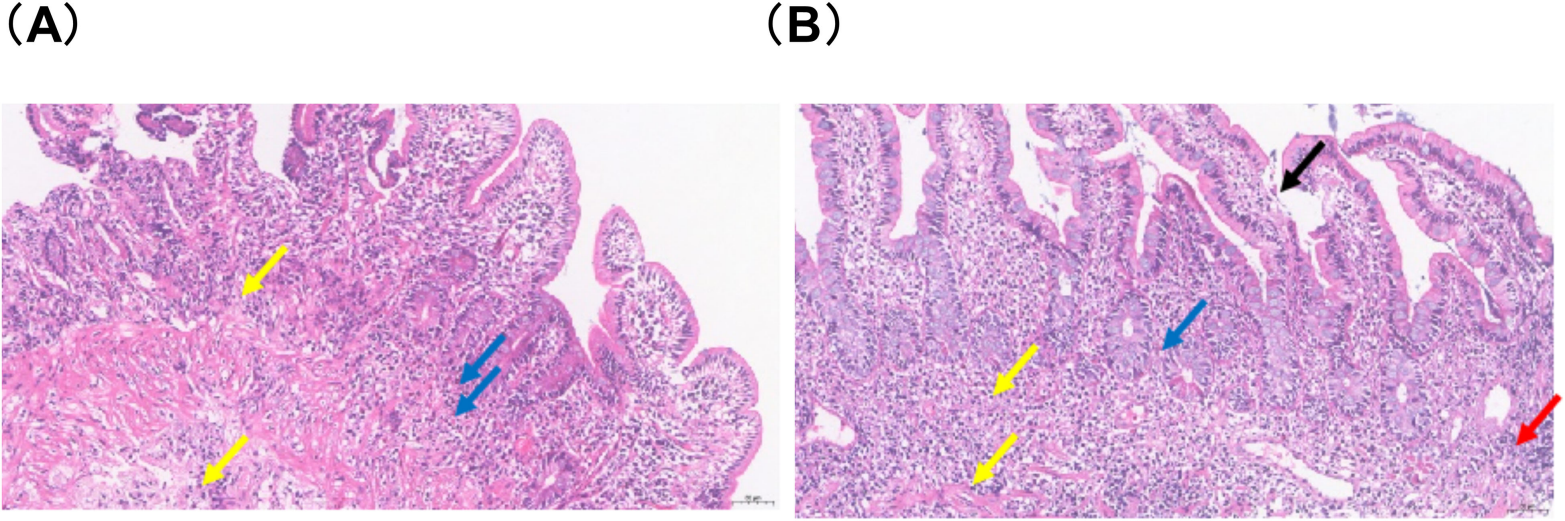
Extensive ulcer formation in intestinal tissues. **(A)** Significant lymphocyte and macrophage infiltration (yellow arrow) and eosinophil infiltration (blue arrow) in the lamina propria and submucosa (H&E ×200, Scale bar = 50 μm). **(B)** Extensive ulceration; columnar and goblet cells visible in villous epithelium, with partial villous epithelial shedding and loss (black arrow). Necrotic cell debris (red arrow), lymphocyte and macrophage infiltration (yellow arrow), and eosinophil infiltration (blue arrow) visible in the lamina propria and submucosa (H&E ×200, Scale bar = 50 μm).

Electromyography and nerve conduction studies showed myogenic damage and peripheral nerve damage (sensory fibers) in the upper and lower limbs. Biopsies from the duodenum, jejunum, and gastric antrum demonstrated eosinophil-rich chronic mucosal inflammation and associated intraepithelial neoplasia (**Figure 3A**). Colonoscopy biopsy showed sigmoid colon polyp resection, with biopsy revealing chronic mucosal inflammation and EOS in the terminal ileum, cecum, and transverse colon (**Figure 3B**).

Lymph node biopsy revealed 15 EOS per high-power field, with negative tuberculosis PCR testing. PET-CT showed multiple enlarged lymph nodes throughout the body, the largest measuring 2.3×2.5 mm, with partial metabolic enhancement. Urinalysis, urine immunofixation electrophoresis, ANCA, ANA profile, autoimmune liver antibodies, antiphospholipid antibodies, MM-related fluorescence *in situ* hybridization (FISH), flow cytometry, chromosomal analysis, hematologic tumor gene mutation analysis, gene rearrangement, bone marrow flow cytometry, magnetic resonance cholangiopancreatography, whole-body bone scan, bone marrow NGS, and whole-exome sequencing results were all unremarkable.

**Figure 4:**
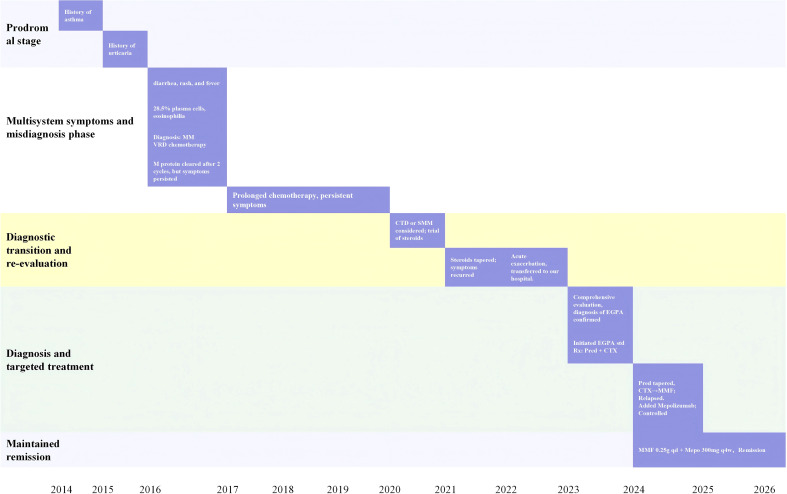
Flowchart of the patient’s clinical course.

Considering that the patient had previously shown M protein in routine bone marrow tests, but bone marrow biopsy did not reveal typical diffuse infiltration of clonal plasma cells characteristic of MM, and there was no hypercalcemia or osteolytic lesions, the diagnosis of MM was uncertain. Combined with the patient’s 9-year history of asthma, sinusitis, neurological damage, recurrent elevation of peripheral blood EOS and EOS%, and eosinophilic infiltration in multiple tissues including the liver, gastrointestinal tract, bone marrow, and lymph nodes, along with improvement during corticosteroid treatment, the diagnosis of EGPA was established based on the 2022 EULAR/ACR EGPA classification criteria (6). The BVAS score (7) was 29, and the FFS score (8) was 1. The induction treatment regimen included “prednisone 50 mg orally once daily (reduced by 5 mg weekly) combined with cyclophosphamide (CTX) 0.5g intravenous infusion every two weeks for six cycles”, along with antibiotic prophylaxis for Pneumocystis jirovecii pneumonia (sulfamethoxazole/trimethoprim 480 mg, three times weekly). After 12 weeks of follow-up (adjusted to prednisone 10 mg orally once daily and mycophenolate mofetil (MMF) 0.5 g orally twice daily), the patient did not experience recurrence of diarrhea, rash, fever, or asthma, and joint pain had resolved. Peripheral blood eosinophil levels gradually returned to normal without an upward trend, hemoglobin stabilized at 115–125 g/L, creatinine remained at 85-95 μmol/L, serum protein electrophoresis did not detect M protein, immunofixation electrophoresis was negative, serum free light chain κ/λ ratio remained stable at 1.1-1.9(reference range: 0.26-1.65), and FISH results remained negative. At 14 weeks of maintenance therapy, the patient gradually developed rashes on both upper limbs, accompanied by watery diarrhea 4–5 times/day, occasional asthma attacks, fatigue, and pain in both hands and shoulder joints. The FFS score was 1, and the BVAS score was 11. Due to poor treatment response, mepolizumab was added to the regimen, and the maintenance treatment plan was adjusted to “prednisone 10 mg orally once daily+MMF 0.5 g orally twice daily+mepolizumab 300 mg subcutaneous injection every four weeks”. At 24 weeks of follow-up, the regimen was adjusted to prednisone 7.5 mg once daily, mycophenolate sodium 0.25 g twice daily, and mepolizumab 300 mg subcutaneous injection every four weeks. The patient’s symptoms of rash, diarrhea, and joint pain had completely resolved, and follow-up chest CT showed significant reduction in liver, spleen, and lymph node size. Bone marrow aspiration and flow cytometry revealed normal hematopoietic function without plasma cell or eosinophil infiltration, and no light chain restriction expression. At 56 weeks of follow-up, the patient was on prednisone 5 mg once daily, mycophenolate sodium 0.25 g once daily, and mepolizumab 300 mg subcutaneous injection every four weeks. Gastrointestinal endoscopy showed resolution of mucosal inflammation without EOS infiltration. At 78 weeks of follow-up, prednisone was discontinued, and the disease remained stable without recurrence of diarrhea, rash, fever, asthma, or joint pain. Peripheral blood EOS counts remained stable at 0.11-0.26×10^9^/L (EOS% 1.5%-3.0%), BVAS score remained at 0, and FFS score was 1.The patient’s clinical course is outlined in **Figure 4**.

## Discussion

EGPA is a necrotizing vasculitis involving small vessels that can affect almost all organ systems. EGPA is typically divided into three stages: the prodromal stage, characterized by asthma and/or allergic rhinitis; the tissue infiltration stage, marked by peripheral eosinophilia and multi-organ eosinophilic infiltration; and the vasculitis stage, accompanied by systemic symptoms such as fever, fatigue, and weight loss (9). This patient clearly experienced all three stages, with asthma and rash appearing earlier than the initial diagnosis of MM. At presentation, the patient exhibited elevated EOS levels during almost every follow-up, and eosinophilic infiltration was observed in all biopsy tissues, along with systemic symptoms such as fever and weight loss. Additionally, the patient had sinusitis and multiple peripheral neuropathies, ultimately leading to the diagnosis of EGPA.

It is noteworthy that two years after the diagnosis of asthma and urticaria, an external hospital diagnosed MM based on the presence of M protein in the bone marrow, biclonal IgG+κ, IgG+λ, and an abnormal increase in immature and mature plasma cells. However, the treatment response was markedly different: after two cycles of MM-specific chemotherapy, M protein disappeared, and plasma cell proportions decreased, but symptoms such as asthma, rash, and diarrhea persisted and were difficult to control. In contrast, standardized EGPA treatment significantly improved these symptoms. This perplexing clinical course necessitates a critical reappraisal: does the presentation align with Occam’s Razor or Hickam’s Dictum? Based on the long-term follow-up, the initial diagnosis of MM appears tenable only on the surface and lacks definitive clonality evidence. First, the patient’s pathological and immunophenotypic results do not align with MM. Bone marrow biopsy did not reveal the hallmark feature of MM—diffuse infiltration of clonal plasma cells—and the immunophenotype lacked typical MM markers such as CD38^+^, CD138^+^, CD56^-^expression, and single light chain restriction. Second, the patient did not exhibit characteristic end-organ damage associated with MM. Third, the MM chemotherapy regimen temporarily reduced M protein levels but failed to control symptoms, whereas standardized EGPA treatment achieved simultaneous resolution of symptoms and plasma cell abnormalities. Furthermore, MM is a malignant plasma cell proliferative disorder with an average survival time of 3–5 years (10), characterized by monoclonal plasma cell proliferation, with biclonal proliferation being rare. Localized MM patients have a 5-year survival rate of 74.8%, while systemic MM patients have a 5-year survival rate of 52.9% (11), with higher recurrence rates correlating with shorter survival times. M protein typically persists, and complete remission after chemotherapy is rare. This patient underwent repeated chemotherapy over seven years with poor efficacy but survived without significant impact.

To clarify the cause of M protein abnormalities, we considered differential diagnoses for diseases that could lead to elevated M protein. For example, POEMS syndrome, whose core diagnostic criteria include polyneuropathy, organomegaly, endocrine abnormalities, M protein, and skin changes, was excluded due to the absence of organomegaly and endocrine abnormalities in this case. Similarly, monoclonal gammopathy of undetermined significance (MGUS) was excluded: MGUS is characterized by asymptomatic M protein elevation without organ damage, whereas this patient exhibited biclonal proliferation, M protein associated with symptoms such as asthma and rash, and symptoms directly related to EGPA organ involvement, fundamentally differing from MGUS. Additionally, given the patient’s multi-system involvement and hepatosplenomegaly, we considered IgG4-related disease but excluded it due to the absence of storiform fibrosis, obliterative phlebitis, and IgG4-positive plasma cell infiltration (≥10/HPF) in biopsy specimens, as well as normal serum IgG4 levels. After ruling out these diseases, we explored whether EGPA could lead to plasma cell proliferation and M protein abnormalities. A literature search using keywords such as “MM”, “plasma cell proliferative disorders”, “EGPA”, and “eosinophils” did not reveal clear reports of EGPA coexisting with MM. Ciftci S et al. (4)described a case of EGPA presenting with polyneuropathy that simultaneously met monoclonal MM diagnostic criteria and responded well to corticosteroid treatment, but the relationship between the two diseases remains unclear. Lee HS et al. (5)reported a case of a 31-year-old male with biclonal MM, significant EOS, and tissue eosinophilic infiltration but no history of asthma or rash, who recovered after chemotherapy and autologous stem cell transplantation.

In the pathogenesis of EGPA, EOS are found to be abnormally activated, primarily driven by cytokines such as interleukin (IL)-5, IL-13, and granulocyte-macrophage colony-stimulating factor, which promote eosinophil maturation. Since these cytokines are driven by Th2 cells, Th2 cells are considered a key component in EGPA pathogenesis (12). Additionally, activated EOS can promote Th2 cells to secrete polarized cytokines such as IL-4 and IL-13, and IL-4 release can accelerate B cell differentiation into IgM plasma cells (13). *In vitro* experiments have shown that EOS can enhance the proliferation of specific malignant plasma cells, promoting the biological characteristics of MM. Co-culture of KAS-6/1, DP-6, and KP-6 with EOS can promote the proliferation of human myeloma cell lines (14). We attempted to identify genetic links between these two diseases through whole-exome sequencing, but unfortunately, no gene mutations associated with EGPA, MM, or other diseases were found in this case. Finally, we hypothesize that the proliferation of EOS following EGPA onset may affect the bone marrow hematopoietic microenvironment, acting on plasma cells through Th2 and other cytokine pathways, leading to pseudo-malignant plasma cell proliferation and M protein production.

Since eosinophilia can also be seen in other diseases, further differential diagnosis is needed to clarify its cause and solidify the diagnosis of EGPA. First, the patient exhibited typical EGPA symptoms, ruling out primary hypereosinophilic syndrome. Second, many solid tumors are associated with eosinophilia, such as skin, gastrointestinal, breast, and adrenal tumors, while hematologic malignancies often present with secondary eosinophilia, including eosinophilic leukemia, acute myeloid leukemia, chronic myeloid leukemia, myelodysplastic syndrome, and other myeloproliferative neoplasms (15). Although MM was initially suspected, bone marrow examination did not reveal malignant clonal infiltration, and cytogenetic/molecular biological tests did not detect malignant clones. The slow disease progression was inconsistent with the aggressive nature of hematologic malignancies, leading to its exclusion. Allergic diseases may only cause localized eosinophilia without systemic vasculitis manifestations. Infectious diseases require evidence of exposure and positive pathogen testing, which were absent in this case, leading to their exclusion.

## Conclusion

As a rare disease, EGPA can affect various systems. When the bone marrow is involved, plasma cell proliferative disorders such as MM may occur, leading to related clinical manifestations. It is essential to carefully elucidate their relationship, prioritize the principle of parsimony (Occam’s razor) in diagnosis, and conduct comprehensive multi-system evaluations and rigorous differential diagnoses to avoid delayed diagnosis and treatment.

## Data Availability

The original contributions presented in the study are included in the article/supplementary material. Further inquiries can be directed to the corresponding author.
